# The effect of the mechanodynamic lung environment on fibroblast phenotype via the Flexcell

**DOI:** 10.1186/s12890-024-03167-7

**Published:** 2024-07-27

**Authors:** S Al Yazeedi, A. F Abokor, J Brussow, F Thiam, S Phogat, E.T. Osei

**Affiliations:** 1 Department of Biology, University of British Columbia - Okanagan Campus, 3187 University Way, ASC366, Kelowna, BC V1V1V7 Canada; 2 Centre for Heart Lung Innovation, St. Paul’s Hospital, Vancouver, BC V6Z 1Y6 Canada

**Keywords:** Lung fibroblasts, Mechanical lung environment, Flexcell tension bioreactor, Strain amplitudes, Frequency, In vitro models, Cyclic and static strain

## Abstract

The lung is a highly mechanical organ as it is exposed to approximately 10^9^ strain cycles, (where strain is the length change of tissue structure per unit initial length), with an approximately 4% amplitude change during quiet tidal breathing or 10^7^ strain cycles at a 25% amplitude during heavy exercises, sighs, and deep inspirations. These mechanical indices have been reported to become aberrant in lung diseases such as acute respiratory distress syndrome (ARDS), pulmonary hypertension, bronchopulmonary dysplasia (BPD), idiopathic pulmonary fibrosis (IPF), and asthma. Through recent innovations, various in vitro systems/bioreactors used to mimic the lung’s mechanical strain have been developed. Among these, the Flexcell tension system which is composed of bioreactors that utilize a variety of programs in vitro to apply static and cyclic strain on different cell-types established as 2D monolayer cultures or cell-embedded 3D hydrogel models, has enabled the assessment of the response of different cells such as fibroblasts to the lung’s mechanical strain in health and disease. Fibroblasts are the main effector cells responsible for the production of extracellular matrix (ECM) proteins to repair and maintain tissue homeostasis and are implicated in the excessive deposition of matrix proteins that leads to lung fibrosis. In this review, we summarise, studies that have used the Flexcell tension bioreactor to assess effects of the mechanical lung on the structure, function, and phenotype of lung fibroblasts in homeostatic conditions and abnormal environments associated with lung injury and disease. We show that these studies have revealed that different strain conditions regulate fibroblast proliferation, ECM protein production, and inflammation in normal repair and the diseased lung.

## Introduction

During respiration, the respiratory volume changes rhythmically due to the movement of the chest wall and diaphragm [1, 2]. These changes subject the lungs to varying degrees of mechanical forces, creating a mechanodynamic environment important for lung tissue homeostasis [3, 4]. Hence, the lung is a highly mechanical organ and is exposed to approximately 10^9^ strain cycles (where strain is the length change of a structure per unit initial length) with an approximately 4% amplitude change during quiet tidal breathing [2]. The lungs are also exposed to 10^7^ strain cycles with a 25% amplitude change during heavy exercises, sighs and deep inspirations in its lifetime [2]. This mechanical breathing environment means lung tissue is constantly subjected to dynamic mechanical stimuli, which affect cellular phenotype through mechanotransduction signalling pathways [5]. In the healthy breathing lung, these pathways are important for tissue homeostasis and immune protection [6]. However, recent studies assessing different mechanical forces in respiratory diseases have shown, 1. pulmonary cell phenotype and morphology changes, 2. defective pulmonary inflammatory responses, and 3. fibrotic responses, through lung cell proliferation and increased production of extracellular matrix (ECM) proteins in the abnormal mechanodynamic lung environment [6–8].

The pulmonary cells affected by the mechanodynamic lung environment include but are not limited to different immune cells, the airway and alveolar epithelium, endothelial cells, airway smooth muscle cells and fibroblasts [9]. Of the lung mesenchymal cells impacted by the mechanical environment, pulmonary fibroblasts form a major part of lung’s airway and alveolar tissue [10] and are the main effector cells responsible for producing ECM proteins that make up the lung’s structure [11, 12]. Added to this, the involvement of lung fibroblasts in inflammatory responses have also been highlighted [11–14]. These functions and phenotypes of lung fibroblasts are regulated by the lung’s mechanical environment via direct control of cellular characteristics including proliferation rates, ECM protein, growth factor, cytokine and chemokine mediator production among others and is essential for maintaining tissue homeostasis [2]. In different lung diseases however (e.g., acute respiratory distress syndrome (ARDS), pulmonary hypertension, bronchopulmonary dysplasia (BPD), ventilation induced injury (VILI) and asthma), the dysregulated mechanical lung environment causes fibroblasts to adopt a defective phenotype which may play important roles in disease pathogenesis [15].

ARDS is a severe lung condition that results from infection, injury, or trauma, and leads to the accumulation of fluid in the lung alveoli, resulting in dyspnea [16]. Pulmonary hypertension is characterised by elevated lung arterial blood pressure, excessive/abnormal cellular proliferation, and apoptosis rates [17–19]. BPD occurs in premature infants who require mechanical ventilation and oxygen therapy due to damaged and under-developed lungs which leads to increased lung tissue scarring and stiffness [20, 21]. In matured patients of all ages, ventilator-induced lung injury (VILI) characterised by lung inflammation, fluid accumulation, tissue fibrosis and damage also occur after prolonged use of mechanical ventilation [22, 23]. In asthma, allergic and non-allergic triggers cause eosinophilic or neutrophilic and T_H_2 inflammation as well as airway bronchoconstriction that leads to wheezing, shortness of breath, and chest tightness [24–26]. As part of the general mechanisms of these diseases, there is a major defective tissue repair component with lung fibroblasts playing a central role [13, 14]. This is also seen in idiopathic pulmonary fibrosis (IPF) which is a progressive interstitial disease characterized by excessive fibrosis, cellular proliferation, and tissue remodeling as well as the formation of fibroblastic foci in the lung parenchyma [27, 28]. Although studies and reviews have focused on the biochemical characteristics of lung fibroblasts in all these diseases, less attention has been paid to the interaction between the abnormal lung mechanical environment and defective lung fibroblast phenotype.

The various forces encountered in the lung’s mechanical environment include cyclic uniaxial and equibiaxial strain (meaning strain in one or two directions or axes), compression forces, shear stress, changing gradients of tissue stiffness etc. [29]. In addition to complex biomimetic systems such as microfluidic lung-on-chips and 3-dimensional (3D) hydrogel models that assess shear stress and stiffness, characteristic in vitro systems that mimic cyclic uniaxial, biaxial, and equibiaxial dynamic strain loading include, compressors, as well as static and cyclic vacuum pressure-controlled bioreactors [30]. Since the early 90’s, a variety of complex in vitro systems or bioreactors have been established and improved to enable the study of the lung mechanical environment’s effect on cellular phenotype [30, 31]. These systems have included the earliest versions of mechanical bioreactors that employed solenoid units, to current vacuum pressure-controlled bioreactors [25]. Of these, the commercially available Flexcell system which is a static and cyclic vacuum-controlled bioreactor is widely utilised [32] and provides a standardised and optimized model for static and dynamic strain application in lung research [31].

Though some reviews on the effects of lung biomechanics on different cells exist, these have mostly presented a summary or a survey of studies that use different mechanical systems to assess a variety of pulmonary cells such as the epithelium, the airway smooth muscle and immune cells [7, 29, 31, 33–35]. Giving the importance of lung fibroblasts in driving abnormal tissue repair mechanisms in lung diseases, and the availability of studies specifically using the Flexcell to assess the effect of different strain environments on fibroblast phenotype, this review explores the unique opportunity to critically examine multiple experiments across different studies that assessed (abnormal) lung mechanodynamic regulation of lung fibroblast phenotype. The findings of this review unlike others that focus on different cell-types and a variety of mechanical systems, will therefore serve to provide a clear overview of the mechanisms through which lung fibroblasts contribute to and are affected by the abnormal mechanical lung environment in health and disease. Hence, we focus on studies with the Flexcell bioreactor and provide a summary of findings on how the lung’s mechanical environment regulates fibroblast phenotype and function in homeostasis, during injury, and disease.

## Methods of applying strain on pulmonary cells

In vitro bioreactors established to mimic cell-stretching and straining in vivo gained traction in the early 90’s with systems such as the bioreactor established by Skinner and colleagues where a solenoid unit was used to generate an electromagnetic field that extended one side of fetal rat lung cells grown on a Gelfoam [36, 37]. In another study, alveolar type II (ATII) cells were seeded and stretched on hydrostatic pressure-controlled flexible poly(dimethylsiloxane) (PDMS) models [38]. However, both the solenoid Gelfoam and PDMS hydrostatic models produced non-uniform strain conditions and varying effects on cell phenotypes [31]. Another bioreactor that was developed to apply a more uniform equibiaxial strain utilised an annular indentor to deform flexible silastic membranes [39]. This system enabled the continuous application of a predictable uniform strain for 1 hour, enabling stable quantifiable measurements of alveolar responses to be made [39]. The indentor system, together with the commercially available Flexcell system, and STREX Incorporated’s stretching apparatus (both of which have similar principles of operation as discussed in the next section) have been the main bioreactors employed in biomechanical studies to assess the effect of strain on cells [31]. In pulmonary research, there is also the moving air–liquid interface (MALI) bioreactor that consists of a basal and apical chamber separated by a stretchable, porous membrane on which airway or alveolar epithelial cells are cultured at an air–liquid interface (ALI) that allow epithelial cell differentiation into surfactant-producing alveolar epithelium or pseudo-stratified mucus-producing airway epithelium as can be found in vivo [40]. The flexible membrane is strained by regulating the air column on top of the apical chamber to mimic breathing [31, 40]. As a porous membrane is required for ALI culture and subsequent epithelial differentiation, the MALI system offers advantages over other currently used bioreactors which employ non-porous silicone membranes when studying pulmonary epithelial cell biology [31, 40]. It is envisaged that improvements and further developments in these systems will enable the establishment of complex physiologically relevant multicellular models to closely mimic the pulmonary environment. In line with this, microfluidic gut-on-chip systems have been developed where lung epithelial-ALIs on stretchable PDMS membranes are co-cultured with endothelial cells, before applying uniaxial strain to pressure chambers beside the membrane with the aid of vacuum provided by the commercially available Flexcell [41, 42]. There are also precision cut lung slices (PCLS) where lung tissue explants (with the native tissue components and complexity), cut to thin sections of 200 – 500µm are cultured in media and stretched through pressure application [43].

Reviews assessing how the different bioreactors outlined have assessed the pulmonary environment’s effect on cell types including epithelium, endothelial and airway smooth muscle cells exist [7, 29, 31, 33–35]. However, there is a paucity of reviews on studies investigating lung fibroblast function in the mechanical lung. Here, the commercially available Flexcell bioreactor with standardised/optimised (2D) protocols and cell-embedded 3-dimensional (3D) hydrogel models that mimic fibroblast-embedded ECM have been used. There is therefore merit in assessing studies on lung fibroblast function using the Flexcell to enable the comparison of data from the same equipment in different experiments conducted across different research. This also provides a reference for future mechanical lung research on fibroblasts and other lung mesenchymal cells.

## The Flexcell system

The Flexcell Tension Systems are composed of computer-controlled bioreactors that use a variety of programs in vitro to apply static and cyclic strain on different cell-types established as 2D monolayer cultures or cell-embedded 3D hydrogel models, to enable the assessment of cellular response to mechanical strain [32, 44]. These systems enable the examination of biochemical and phenotypical changes to cells because of their mechanical environment such as those experienced in the dynamic lung tissue [32, 45, 46]. In the pulmonary field, the Flexcell has aided in simulating the normal breathing environment and the abnormal mechanical environment experienced in chronic diseases (e.g., ARDS, asthma) [47].

**Fig. 1:**
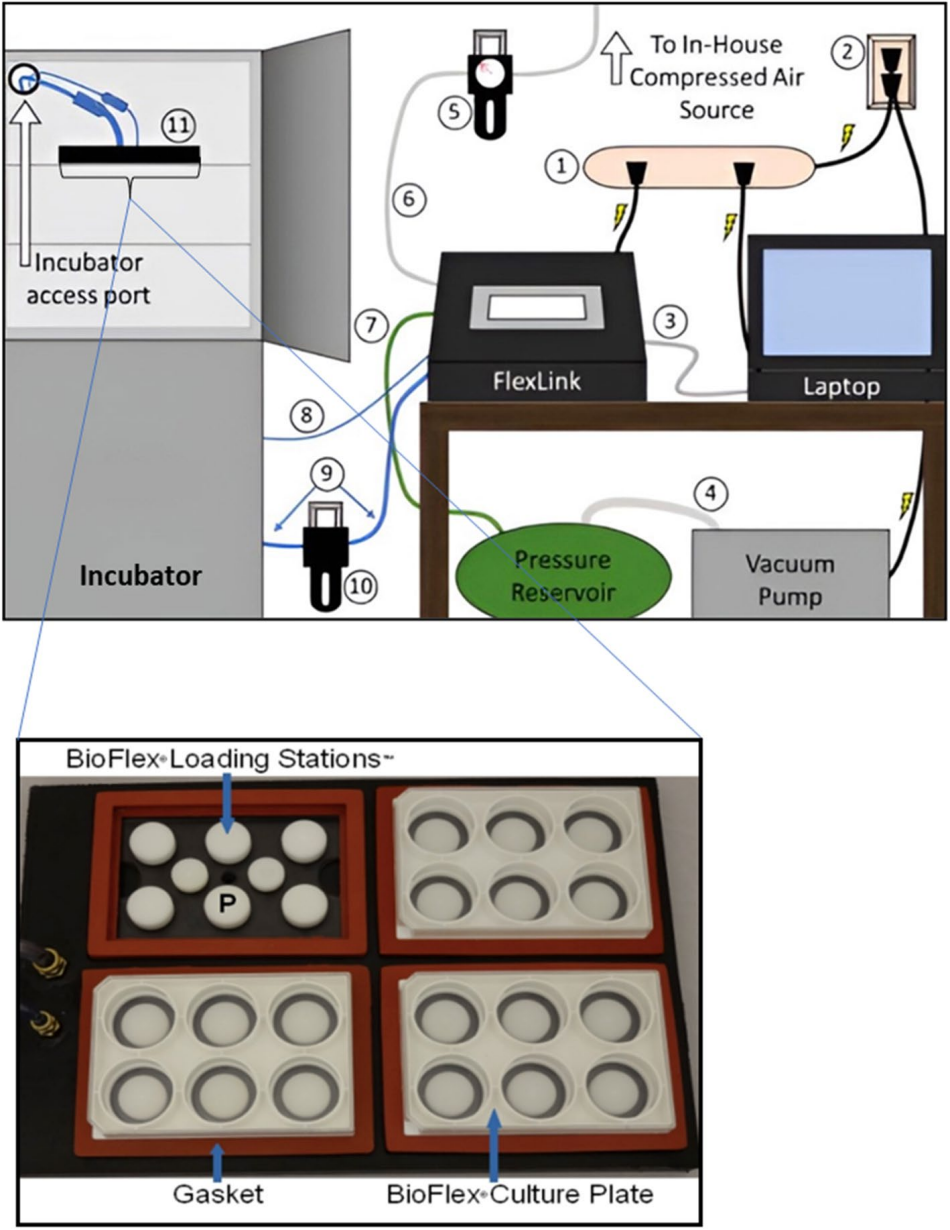
Set up of Flexcell Tension Systems. Top—Schematic of the Flexcell® FX-6000™ System, in order of functionality 1–11. “1) Surge protected power strip, 2) power outlet, 3) Ethernet cable, 4) reinforced vacuum tubing, 5) compressed air regulator/filter, 6) vent tubing, 7) system tubing, 8) flex in tubing, 9) flex out tubing, 10) water trap, 11) baseplate”. Bottom- An enlargement of a top view of the baseplate in which the different culture plates sit for strain experiments showing white loading posts. Adapted from Flexcellint® with permission

**Fig. 2:**
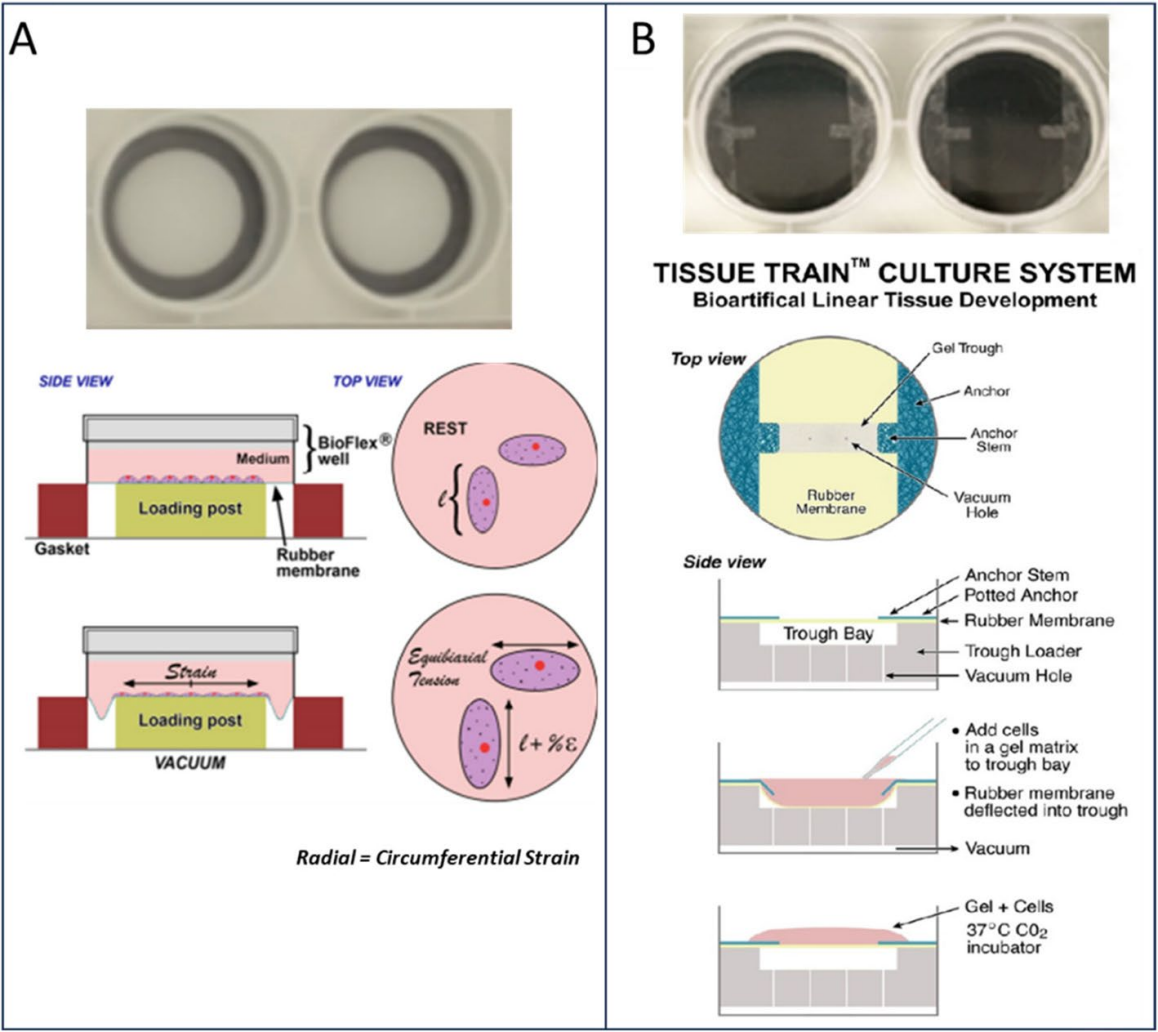
Bioflex and Tissue Train in vitro models of the Flexcell system. **A** Image of two wells from the 6-well Bioflex culture plate with a flexible membrane bottom and a schematic showing a 2D monolayer of cells grown in the plate across a cylindrical loading post creating equibiaxial tension. **B** Image of two wells out of the 6-well Tissue Train culture plate with a top view schematic of a cell-embedded gel construct connected to the anchor stems, the side view illustrates that when vacuum is applied, the rubber membrane deforms downwards to create space for the 3D linear gel to form. Adapted from Flexcellint® with permission

The Flexcell Tension system has had different versions over the years including the FX-3000™, FX-4000™, FX-5000™, and more recently the FX-6000™ (Fig. 1). These function by applying a specified vacuum under static or cyclic control to deform a flexible membrane of a culture plate on which cells grow either in a 2D monolayer or embedded in 3D collagen-I-gels (Fig. 2) [48, 49]. To mimic the mechanodynamic environment, the Flexcell functions by applying vacuum at frequencies ranging from 0.01 to 5Hz, yielding maximal substrate amplitude elongation of up to 33% to generate deformation of 2D and 3D cell models via flexible-bottom culture plates [48, 49]. Strain frequencies can also be indicated in cycles per minute (cpm) where 6 and 300 cpm are equivalent to 0.01 and 5Hz respectively [48, 49]. Mechanical strain generated by the Flexcell can be programmed into different waveforms, with different frequencies (in cpm or Hz), and elongation amplitude percentages, which enables the mimicking of different mechanodynamic organs in the body including the heart and lungs.

**Fig. 3:**
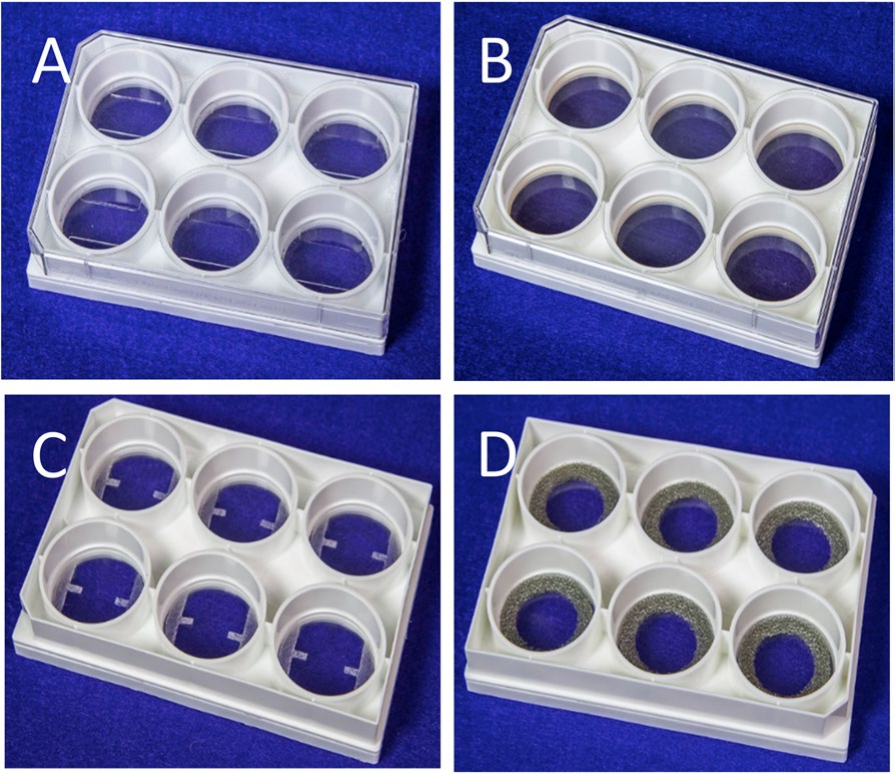
Images of 4 different 6-well Flexcell culture plates. **A** Uniflex culture plate. **B** Bioflex culture plate. **C** Tissue Train Linear culture plate. **D** Tissue Train Circular Foam culture plate. Adapted from Flexcellint® with permision

The application of strain to 2D and 3D cell models in the Flexcell is made possible through flexible-bottom specialised cell culture plates. Specialised plates known as the UniFlex (Fig. 3A) and BioFlex (Fig. 3B) are used for 2D monolayer cell cultures. The UniFlex plates strain cells in a uniaxial vector where the force acts in one direction or axis, whereas the BioFlex strains cells equibiaxially where uniform radial and circumferential strain in two directions or axes are applied [50, 51]. 3D collagen-I-embedded cell models on the other hand are created in tissue train culture plates which come in two forms: 1) tissue train linear plates (Fig. 3C) where cell-embedded hydrogels are established and attached to 2 matrix bondable cerex nylon mesh or urethane polyester foam anchor tabs and, 2) the tissue train circular foam plates (Fig. 3D) which have matrix bondable foam anchors. Just as with 2D plates, the tissue train linear gels apply uniaxial strain while tissue train circular gels apply equibiaxial strain. Table 1 summarises various culture plates and their corresponding models. In the following sections, we will assess how the Flexcell, and its specialised plates have been used to study different mechanodynamic cell models and their use in understanding fibroblast phenotype and function in the healthy, injured, or diseased lung models.

**Table 1:** Type of specialised Flexcell plate, strain vector and in vitro model created

Culture Plate Style	Strain Vector	Models
UniFlex	Uniaxial	2D Monolayer cell culture
BioFlex	Equibiaxial	2D Monolayer cell culture
Tissue Train Linear	Uniaxial	3D Tissue culture
Tissue Train Circular Foam	Equibiaxial	3D Tissue culture

## Fibroblast models in the Flexcell assessing tissue homeostasis in health and remodelling in diseases

### Cellular proliferation

The mechanodynamic lung environment has been shown to regulate fibroblast proliferation in the healthy lungs and during lung injury associated with different disease phenotypes [52]. In line with this, various mechanical flexcell models are used to mimic lung diseases, and features of injured lungs [53–57]. These have used continuous human fibroblast secondary cell-lines and animal-derived lung fibroblasts to demonstrate how mechanical forces regulate fibroblast proliferation while mimicking different pathologic characteristics of lung injury and disease [53–57].

In line with studying the effects of healthy and abnormal mechanical strain in the lung, Xie *et al.*, employed the human fetal lung fibroblast MRC-5 cell-line to assess the role of the mechanical environment in ARDS. To do this, MRC-5 lung fibroblasts were seeded as 2D monolayers on BioFlex plates, exposed to different concentrations of lipopolysaccharide (LPS) and strained via the FX-5000™ at a frequency of 0.1 Hz with amplitudes of 5%, 10%, 15% and 20% for 48 hours [53]. It was found that with or without LPS, amplitudes of 5 and 10% caused increased proliferation rates of MRC-5 lung fibroblasts while higher amplitudes of 15% and 20% led to decreased proliferation rates [53]. This study showed that in ARDS, increased dynamic strain loads may be enough to decrease fibroblast proliferation rates and thus tissue repair mechanisms in the presence of bacterial infections as mimicked by LPS, a component of the bacterial cell wall [53, 58]. In addition, Bishop *et al.*, used the human fetal lung fibroblast IMR-90 cell line to establish a 2D model in the Flexcell and evaluated how increased pulmonary mechanical load might contribute to cellular hypertrophy and hyperplasia in lung pathologies or diseases [54]. IMR-90 lung fibroblasts were exposed to a strain amplitude of 10% at a frequency of 1 Hz, for up to 5 days which caused a 39% increase in cell numbers (above the control) on day 2, and a 163% increase after 4 days [54]. The ability of mechanical strain to stimulate cellular proliferation was associated with the production of soluble mitogens as measured in the supernatant medium from strained fibroblasts which was linked to possible mechanotransduction pathways including an increase in inositol triphosphate, diacylglycerol and prostaglandin E2 signaling [59]. Hence these studies using human fetal lung fibroblast cell lines (MRC-5 and IMR-90) provided models to mimic healthy (strain amplitudes ~ 5%) and abnormal (strain amplitudes ~ 20%) mechanoenvironments to mimic abnormal features such as hyperplasia and hypertrophy or disease conditions (e.g., ARDS), and found that lower amplitudes which may simulate healthy environments promotes cell proliferation while higher strain amplitudes that may mimic abnormal conditions caused a decrease in cell proliferation.

Other studies used primary animal-derived fibroblasts to assess the role of cellular proliferation in the healthy and pathological lung’s mechanical environment. Sanchez-Esteban *et al.*, studied the role of intermittent stretch in late fetal lung development by culturing fetal rat lung fibroblasts isolated at different gestational ages as a monolayer on BioFlex plates and applied a 5% cyclic stretch at 60 cpm intervals for 24 hours with the FX-3000™ [56]. This setup mimicked the mean hourly human fetal breathing rates and caused a decrease in cellular proliferation by 42% when compared to unstrained controls [56]. Decreased proliferation rates was shown to be time-dependent and linked to increased apoptosis as well as a characteristic arrest in the G_0_/G_1_ phase of the cell cycle in lung fibroblasts from gestational day 19 compared to day 18 [56]. This data demonstrated the importance of mechano-temporal regulation in lung development. In another study, fetal rat lung fibroblasts were used to investigate the role of the epidermal growth factor receptor (EGFR) in stretch-dependent mechanical lung injury as a result of ventilator-induced lung injury (VILI) linked to bronchopulmonary dysplasia (BPD) [57]. Fetal rat lung fibroblasts with the EGFR gene present (wild-type) or knocked out (EGFR knockout) were cultured as 2D models on BioFlex plates and mechanically strained for 48 hours at a frequency of 40 cpm with a 2.5% amplitude to mimic physiological conditions, and a 20% amplitude to simulate lung mechanical injury [57]. It was found that the expression of proliferating cell nuclear antigen (PCNA), a DNA-associated protein and marker for cell proliferation in wild-type fibroblasts was reduced by 71% after the 20% amplitude strain compared to 2.5% strain [57]. However, in EGFR knockout lung fibroblasts, there was no significant change in cellular proliferation rates comparing different strain environments. Further although 20% strain caused increased apoptosis of wildtype mouse lung fibroblasts, EGFR knockout protected fibroblasts from strain induced cell death [57]. These findings point to an EGFR-dependent regulation of lung fibroblast proliferation and apoptotic mechanisms that play a role in potential mechanisms of VILI and BPD [57]. In summary, the data from animal studies using primary rat fetal lung fibroblasts show that low amplitudes of 5% and those as high as 20% caused decreased proliferation rates with potentially higher effects from higher mechanical strains amplitudes. The involvement of growth factor receptors and nuclear co-factors such as EGFR and PCNA in mechanotransduction pathways might provide mechanistic explanations for the increased effects of higher amplitudes (~ 20%) in pathological conditions.

All together, the studies in this section utilised both continuous human fetal lung fibroblast cell-lines and animal-derived primary lung fibroblasts to demonstrate the role of the healthy and defective mechanodynamic environments in regulating lung fibroblast proliferation. Interestingly, models that subjected fibroblasts to amplitudes that mimic physiological strain conditions, led to increased proliferation rates while models that subjected fibroblasts to abnormal strain conditions (~ 20%) potentially encountered in hypertrophy and/or hyperplasia, ARDS, VILI and BPD led to decreased proliferation rates. Increased fibroblast proliferation is essential for tissue repair mechanisms in the healthy lungs while decreased proliferation rates may affect the ability of fibroblasts to effectively repair damaged tissue during tissue regeneration needed to counteract pathological conditions [13, 14, 60].

### Remodelling of the extracellular matrix

In the mechanical lung, fibroblasts are the main cells responsible for ECM protein turnover (e.g., collagen-1, fibronectin, etc.) that constitute the tissue structure [61]. Maintenance of ECM composition and tissue stiffness by fibroblasts is crucial for homeostatic conditions of the healthy lung [62]. However, altered mechanical strain associated with lung injury and disease can cause defective ECM production and remodelling by influencing lung fibroblast expression and deposition of various structural proteins [63]. The Flexcell has enabled the assessment of how the pulmonary mechanical environment regulate fibroblast ECM production in homeostatic conditions and in diseased lung tissue associated with different pathologic conditions [53, 55, 64–66]. In relation to this, Flexcell studies have shown that the mechanical environment regulates ECM protein gene expression by influencing lung fibroblast-dependent release, concentration and activity of growth factors (e.g. transforming growth factor (TGF)-β1) [53, 55, 64–66]. Similar to the previous section, these studies have utilised continuous human cell-lines, and primary human fibroblasts to create mechanical injury models in the Flexcell system [53, 55, 64–66].

Using continuous human lung fibroblast cell lines, Xie *et al.*, modeled the pathology of ARDS by applying different strain amplitudes to MRC-5 lung fibroblasts at a 0.1 Hz frequency with LPS stimulations for 48 hours and measured the concentration of TGF-β1 and collagen-I expression in cells [53]. At an amplitude of 5%, there was no difference in TGF-β1 and collagen-I production [53]. However, a 10% amplitude caused an increase in collagen-I and TGF-β1 mRNA expression, which was notably higher (almost 2x) at 15% and 20% strain amplitudes [53]. The mechanism by which excessive mechanical strain and bacterial infection mimicked with LPS potentially contribute to ARDS may be due to their ability to reconstruct the distribution and structure of cytoskeletal proteins which causes the reorganization of mechanosensitive receptors and affect fibroblast expression of ECM proteins [47]. In line with this, it was determined that high levels of mechanical strain that injures lung tissue result in increased expression of procollagen type I in IMR-90 lung fibroblasts [55]. The level of this increase varied depending on the ECM protein substrate on which lung fibroblasts were cultured [55]. Specifically, when IMR-90 fibroblasts were cultured on laminin and elastin-coated culture plates, a significant increase in procollagen-I expression was observed after cells were strained at a pressure of -13 kPa or 20% amplitude with a frequency of 1 stretch per second for 48 hours [55]. On fibronectin-coated plates fibroblast procollagen-I expression decreased [55]. Corroborating this, it was also found through [^3^H]proline incorporation assays that strained lung fibroblasts on laminin and elastin substrates increased their rates of new procollagen I synthesis while no significant differences were found on fibronectin substrates [55]. In the lungs, laminin is one of the main components of the basement membrane that anchors the epithelium via hemidesmosomes, regulates collagen function and vascular development, and is essential for cellular interaction through integrin binding [67]. Elastin is a major ECM protein in larger vessels and found in the interstitial space of alveolar septal tissue [68]. Fibronectin is essential for the ECM structure together with collagen and forms a major component of the provisional matrix laid down during wound healing [68, 69]. This study therefore showed how important ECM proteins directly involved in structure and load bearing functions such as transmural pressure and longitudinal tension resistance in the lungs also differentially regulate fibroblast-dependent remodeling responses [55]. Suggested mechanisms of these observations involve mechanotransduction signalling potentially linked with integrin-mediated cellular-ECM interactions in the lung’s mechanical environment [13]. Taking together, these studies show that mechanical strain amplitudes of 10% or higher significantly increase the production of TGF-β1-dependent (pro-)collagen-I from lung fibroblasts. Additionally, there is evidence to suggest high strain amplitudes regulate lung fibroblast collagen expression and synthesis and thus ECM remodeling due to specific matrix protein interactions.

To clarify the role of disease-specific effects in the lung mechanical environment’s regulation of fibroblast ECM production, studies have involved the use of isolated primary fibroblasts from healthy and diseased human lungs. Pulmonary fibrosis is a feature of various pulmonary diseases and is characterised by the uncontrolled accumulation of ECM proteins such as collagen I, fibronectin etc. in the lung parenchyma and airways [70]. Blaauboer *et al.*, investigated the role of mechanical strain in the lungs on fibroblast-to-myofibroblast (FMT) differentiation and the expression of various ECM proteins by primary human lung fibroblasts (PHLFs) [64]. PHLFs were cultured as 2D monolayers on BioFlex plates and stimulated with TGFβ1 after which cyclic mechanical strain at a frequency of 0.2 Hz and 10% amplitude was applied using the FX-4000 for 48 hours [64]. Following strain application there was a reduced TGF-β1-dependent differentiation of PHLFs into the highly synthetic myofibroblasts, responsible for increased ECM production in fibrotic conditions [64, 71]. This was evidenced by decreased mRNA expression of α-smooth muscle actin (α-SMA), a cytoskeletal protein and FMT marker as well as tenascin C, collagens Iα1, Iα2, 3α2, and 5α2 [64]. PHLFs were then assessed to determine the involvement of autocrine TGF-β signaling in FMT regulation. It was demonstrated that although TGF-β dose-dependently increased the expression of its own isoforms, mechanical strain on PHLFs inhibited this autocrine signalling by reducing the expression of TGF-β1 and β2 [64]. Mechanical loads in the lung’s ECM is linked the activation of latent TGF-β1 [72]. Hence this study points to a role for physiologic strain conditions in the lung’s breathing environment potentially reducing endogenous TGF-β expression linked to FMT and fibrosis via regulation of its autocrine signaling. In a different study, Ludwig *et al.*, mimicked the effects of the defective mechanical environment in asthma by culturing isolated primary bronchial fibroblasts from asthmatic patients and non-asthmatic controls, as 2D monolayers on BioFlex plates and subjecting them to a 30% biaxial strain at a 1 Hz frequency for 24 hours, using the FX-3000 [65]. After application of maximal strain, there was increased mRNA expression of key proteoglycans, versican and decorin, in asthma-derived compared to non-asthma-derived bronchial fibroblasts [65]. Versican, a large aggregating proteoglycan essential for regulating the lung’s viscoelastic properties and decorin, a small leucine-rich proteoglycan that interact with TGF-β and regulate collagen fibril spacing, have both been implicated in increased bronchial tissue remodeling in asthma [73, 74]. This points to a potential regulatory role of the abnormal mechanical lung environment in the pathogenesis of asthma. The probable mechanism by which high amplitude strain in asthmatic lungs cause increased proteoglycan ECM protein deposition was demonstrated by Le Bellego and colleagues using a similar experimental set-up of asthma- and non-asthma-derived primary bronchial fibroblasts cultured on BioFlex plates, subjected to 30% biaxial strain at 1Hz frequency for 1 and 24 hours [66]. High amplitude strain, mimicking the abnormal mechanical lung environment activated mitogen-activated protein kinase (MAPK) signalling proteins, including an increased phosphorylation of the c-Jun NH2-terminal kinase (JNK) and decreased phosphorylation of extracellular signal-regulated kinase (ERK)1/2, in asthma-derived compared to non-asthma-control bronchial fibroblasts [66, 75]. Phosphorylation of MAPK signalling proteins eventually lead to the downstream activation of transcription factors involved in the expression of ECM proteins [76]. Interestingly, it was found that, in strained asthma-derived bronchial fibroblasts, increased and decreased phosphorylation of JNK and ERK1/2 respectively was also associated with increased expression of the proteoglycans versican and decorin [66] pointing to a direct link with bronchial wall remodeling in asthma. Added to these Sundarakrishnan and colleagues established PHLF embedded 3D rat tail collagen I and dityrosine cross-linked silk collagen I hydrogels in the tissue train linear plates to recreate fibroblastic foci in IPF [77]. 3D hydrogels were established solely embedded with PHLFs or as a triculture where PHLFs were embedded around an engineered endothelialized lumen with the alveolar epithelial A549 cell-line seeded on top [77]. These models were cultured attached to the tissue train linear plate anchors and left in static conditions or strained at a 10% amplitude and 0.2Hz [77]. It was found using confocal and second harmonic generation non-linear optical microscopy (SHG-NLOM) that PHLFs and collagen fibers in static conditions in mono and tricultures aligned parallel to the y axes of the hydrogel and replicated the phenotype of cells in fibroblastic foci after TGF-β1 treatment as evidenced by the expression of protomyofibroblastic (vimentin) and then myofibroblastic markers (vimentin, α-SMA, periostin) [77]. Models established with silk collagen I gels lasted longer than regular rat tail collagen I and the application of strain quickened the appearance of fibroblastic foci markers [77]. The use of TGF-β1 and bleomycin (an anticancer drug and fibrotic agent) on the triculture model led to epithelial injury with loss of epithelial markers including junctional proteins E-cadherin and occludin as well as the epithelial cell adhesion molecule (EpCAM) in addition to myofibroblast differentiation, which was reversed by the antifibrotic drug, pirfenidone and partly by nintedanib [77]. These data proved the utility of the tissue train and Flexcell in mimicking complex lung disease pathology using mechanically tunable hydrogels such as silk collagen I and the potential for establishing multicellular studies. In summary, mechanical strain may regulate FMT with consequences for the development of fibrotic lesions in the mechanical lung environment in pulmonary fibrosis and asthma. Further, while strain amplitudes of 10% may be protective or o FMT or stimulate the formation of fibroblastic foci, strain amplitudes of 30% are enough to stimulate increased ECM proteoglycan protein production through the activation of mechanotransduction pathways (e.g., MAPK/ERK) in asthma-derived lung fibroblasts.

Data from studies reviewed here involving different experimental conditions and sources of lung fibroblasts point to the lung mechanical environment’s ability to regulate growth factor and fibrotic pathways (e.g. MAPK) to control ECM production which potentially plays a tole in tissue remodeling and fibrosis in diseases such as asthma. This regulation is affected by ECM-fibroblast interactions. Further, physiological mechanical strain in the lungs may aid in protecting against fibrotic mechanisms such as FMT and the production of excess ECM proteins.

## Flexcell Fibroblast models assessing airway inflammation

Pulmonary fibroblasts have been shown to not only produce ECM proteins and serve as sentinel cells but also to play an active role in lung inflammation [14, 78, 79]. Furthermore, the pulmonary mechanical environment is known to potentially influence lung fibroblast inflammatory phenotype [57, 80, 81].

Using primary animal lung fibroblasts to understand how mechanical strain regulates inflammation, Hawwa and colleagues cultured fetal mouse lung fibroblasts as 2D monolayers on Bioflex plates, and strained them equibiaxially at 20% amplitude and 40cpm frequency for 48 hours using the FX-4000 [80]. It was shown that there were increased release of pro-inflammatory cytokines and chemokines such as IL-1β, monocyte chemoattractant protein-1 (MCP-1), regulated on activation normal T cell expressed and secreted (RANTES), IL-6, keratinocyte chemoattractant (KC, mouse homologue of IL-8) and tumour necrosis factor (TNF)-α [80]. However, stimulation of fibroblasts with IL-10 before mechanical strain blocked the release of these pro-inflammatory cytokines [80]. Added to this, mechanical strain caused increased lung fibroblast necrosis and apoptosis [80]. The mechanisms possibly involved in inflammatory mediator release was linked to ERK and p38 signaling as well as the activation of the NF-κB transcription factor [79, 82]. In another study to assess the mechanisms of BPD, fetal rat lung fibroblasts cultured as 2D Bioflex models were strained equibiaxially at 2.5% and 20% amplitudes with a 40 cpm frequency for 48 hours using the FX-4000 [57]. The 2.5% and 20% strain amplitudes were chosen to mimic physiological stretch and pulmonary mechanical injury respectively [57]. Fibroblasts used for mechanical strain experiments were either wild type or EGFR knockout cells [57]. In the wild type, macrophage inflammatory protein (MIP-2) and MCP-1 were significantly increased after 20% strain compared to 2.5% strain [57]. However, in the EGFR-knockout lung fibroblasts, 20% strain led to lower MIP-2 release compared to higher MCP-1 concentrations as opposed to 2.5% strain [57]. This data showed that EGFR may be involved in strain-induced lung fibroblast chemokine regulation as part of the mechanisms of BPD. In summary these studies show that 20% mechanical strain amplitudes lead to the increased production of various pro-inflammatory mediators such as IL-1β, RANTES, IL-6, KC (IL-8), TNF-α, MIP-2 and MCP-1 [57]. These inflammatory mediators are all essential for chronic inflammatory conditions pointing to a link between mechanical injury in the lungs and fibroblast-derived inflammation in the abnormal lungs and lung diseases such as BPD.

Further, Hackett and colleagues created a 2D monolayer fibroblast culture model utilising human fetal lung (HFL1) fibroblasts on BioFlex plates, and compared this with linear collagen-I-3D hydrogel models established with the tissue train culture plates [81]. Prior to application of mechanical strain, it was found that there were significantly lower concentrations of pro-inflammatory cytokines IL-6 and IL-8 in the HFL1-collagen-embedded-3D models compared to the Bioflex 2D model [81]. The application of a 1% amplitude stain at 0.2 Hz frequency mimicking 12 breaths per minute for 48 hours to both 2D and 3D models via the FX-5000 did not yield significant changes in the concentrations [81]. This study corroborated others [70] that show a protective effect of physiologic strain amplitudes in the lungs on fibroblast activation and phenotype.

Overall, data from Flexcell experiments show that high strain environments cause the release of inflammatory chemokines and cytokines including IL-1β, RANTES, IL-6, KC (IL-8), TNF-α, MIP-2 and MCP-1 from lung fibroblasts. This was true for lung fibroblasts from different sources (continuous cell-lines primary animal and human lung fibroblasts) and Flexcell frequencies as well as when different conditions were mimicked such as the inflammatory milieu in BPD. Through these studies, mechanisms of mechanical lung regulation of fibroblast phenotype such as how EGFR may be directly involved in regulating mechanically-dependant chemokine/cytokine release from lung fibroblasts is being elucidated. Further, in line with previous studies showing a protective effect of physiologic strain on lung fibroblast ECM production, strain amplitudes mimicking the homeostatic lung environment may be protective of lung-fibroblast derived inflammation. These provide evidence of how the mechanical lung environment may control more than one phenotypic feature of lung fibroblasts in health and disease.

## Further discussion and future directions

The Flexcell bioreactor enables the study of how the mechanical environment affects various pulmonary cells including fibroblasts, the main cells responsible for ECM protein production in the lung. The various studies analysed show the ability of the lung’s mechanodynamic environment to regulate fibroblast phenotype and function such as proliferation rates, ECM production and inflammation as summarised in Table 2. Focusing on the Flexcell across various studies allowed for an unprecedented comparison between experiments using similar set-ups. It is evident from most of these studies that strain amplitudes of ~ 20% to 30% that mimic mechanical injury and the diseased lung generally caused similar effects on fibroblast phenotype including decreased proliferation, increased ECM protein and inflammatory mediator production. Interestingly, this was regardless of the variety of notable contrasts in the details of experimental set-ups with the Flexcell including the range of frequencies used (0.1Hz – 2Hz), duration of experiments (24 – 48 hours) and sources of cells (mainly continuous human secondary cell-lines, primary animal, and some primary human lung fibroblasts). However, unlike the high amplitude strain experiments, amplitudes mimicking the physiologic breathing environment ranging between 1 and 10% caused increased and decreased cell proliferation, inhibited TGF-β dependent increased ECM production and mimicked fibroblast foci in IPF. Apart from the obvious contrasts in the details of the experiments such as the different cellular sources, different studies employed a greater variety of strain amplitudes to mimic the physiologic breathing environment (e.g., 1%, 2.5%, 5%, and 10%). These were chosen from various literature sources where amplitudes as low as 1% are reported to mimic the strain experienced in the fetal lung’s basement membrane [60, 83], 4% amplitude changes are linked to quiet tidal breathing [2], and 10% amplitudes are suggested to fall in the elongation range of the epithelial basement during breathing at 40–100% lung capacity [84]. Interestingly, some of these reported values are based on earlier measurements in the field done in studies with animal models such as rat lungs [84], hence more accurate reference values based on physiologic measurements in humans is needed for future studies.

**Table 2:** Summary of all Flexcell studies assessing the effects of the mechanical lungs on fibroblast phenotype and function

Flexcell study	Amplitude of Strain	Frequency (1 Hz = 60 cpm)	Findings	References
**Cellular proliferation**				
MRC-5 lung fibroblasts 2D culture on BioFlex plates with LPS exposure	5%, 10%, 15%, 20%	0.1 Hz	5% and 10% amplitudes increased MRC-5 proliferation, while 15% and 20% decreased proliferation rates	[53]
IMR-90 lung fibroblasts seeded in a 2D monolayer	10%	1 Hz	IMR-90 cellular proliferation increased by 39% on day 2, and by 163% after 4 days	[54]
Fetal rat lung fibroblasts 2D culture on BioFlex plates	5%	60 cpm	fetal rat lung fibroblasts’ proliferation was decreased by 42%	[56]
Wild-type and EGFR knockout fetal rat lung fibroblasts 2D culture on BioFlex plates	2.5% and 20%	40 cpm	At 20% amplitude the rate of wild-type fetal rat lung fibroblast proliferation was 71% less than the 2.5% amplitude	[57]
**Remodelling of the extracellular matrix**				
MRC-5 lung fibroblast 2D culture on BioFlex plates with LPS exposure	5%, 10%, 15%, 20%	0.1 Hz	No change at 5% amplitude At 10% amplitude, there was an increase in TGF-β1 and collagen-1 production in comparison with unstrained controls At 15% and 20%, TGF-β1 and collagen-1 increased almost twice as much as 10% amplitude concentration	[53]
IMR-90 lung fibroblast seeded on Flex-1 culture plates coated with different ECM proteins (fibronectin, laminin, and elastin)	20%	1 Hz	Expression of pro-collagen type 1 increased on laminin and elastin-coated culture plates On fibronectin-coated culture plates, the expression of pro-collagen type 1 decreased	[55]
PHLFs 2D culture on BioFlex plates with TGF-β exposure	10%	0.2 Hz	Decrease of fibroblast into myofibroblast differentiation This led to a decrease in myofibroblast related genes that induce type-I, type-III and type-V collagen, and tenascin C in PHLFs	[64]
Primary asthmatic and non-asthmatic bronchial fibroblasts 2D culture on BioFlex plates	30%	1 Hz	mRNA expression of proteoglycans such as versican and decorin were higher in asthmatic fibroblasts compared to non-asthmatic fibroblasts	[65]
Primary asthmatic and non-asthmatic bronchial fibroblasts 2D culture on BioFlex plates	30%	1 Hz	Decrease phosphorylation of (ERK)1/2 and increased phosphorylation of JNK in asthmatic fibroblasts compared to non-asthmatic control fibroblasts	[66]
PHLFs solely embedded or embedded surrounding endothelialized lumen with alveolar epithelial cells seeded on top of silk collagen I and rat tail collagen I in tissue train linear plates	Static and 10%	0.2 Hz	TGFβ1 treatment caused protomyofibroblastic and then myofibroblast phenotype in fibroblasts. Cells and collagen fibers aligned parallel to the y axes of the hydrogel. These changes were faster when strain was applied. TGF-β1 and Bleomycin treatment on tricultures caused epithelial injury and fibrotic changes which were reversed by pirfenidone and partly by nintedanib	[77]
**Airway Inflammation**				
Fetal mouse lung fibroblasts 2D culture on BioFlex plates and stimulated by IL-10	20%	40 cpm	Increased pro-inflammatory cytokine concentration such as IL-1β, IL-6, MCP-1, RANTES, KC, TNF-α Stimulation of fetal mouse lung fibroblasts to IL-10 blocked the release of these cytokines	[80]
Wild-type and EGFR knockout fetal rat lung fibroblasts 2D culture on BioFlex plates	2.5% and 20%	40 cpm	The wild-type fibroblast has undergone a significant increase of inflammatory markers MIP-2 and MCP-1 in the 20% amplitude compared to the 2.5% In the EGFR-knockout fibroblasts, MIP-2 concentration was less in the 20% amplitude compared to the 2.5%. And the concentration of MCP-1 was higher in the 20% amplitude compared to the 2.5%	[57]
HFL1 fibroblasts 2D culture on BioFlex plates HFL1 fibroblasts collagen-I-3D hydrogel culture on Tissue Train plates	Static and 1%	0.2 Hz	Prior to mechanical strain, concentration of pro-inflammatory cytokines IL-6 and IL-8 were significantly lower in the 3D hydrogel model compared to the 2D monolayer No significant change in IL-6 and IL-8 after mechanical strain application	[81]

In addition to this, the fact that most of the studies reviewed used continuous secondary cell-lines and primary animal fibroblasts points to a clear gap in the field for more primary human lung fibroblast studies. This is because although continuous cell-lines and primary animal lung cell studies may be suitable and robust for establishing models, performing preliminary studies, and used in combination with specific injury models to mimic diseased lungs, data obtained from these studies are foundational and may not translate directly to clinical or in vivo human conditions. To assess disease-specific effects, it is crucial for future work to widely use primary human lung fibroblasts isolated from healthy individuals and diseased lungs which largely retain and maintain patient-specific phenotypes [13]. Further to this, recent global studies using single cell RNA sequencing has revealed gene markers that can differentiate between different populations of lung fibroblast subtypes such as matrix fibroblasts, lipofibroblasts, myofibroblasts, and other mesenchymal cells such as pericytes and mesothelial cells in lungs [85]. This enables the establishment of unique lung fibroblast subtype 3D in vitro models in the Flexcell that can combine newly identified RNAseq markers with downstream molecular assays to confirm their protein expression and the assessment of cellular phenotype after mechanical strain experiments [86, 87]. These studies will be in line with other global profiling studies involving RNAseq after straining of 2D monoculture leg myoblasts [88] and mass spectrometry proteomics after straining of optic nerve astrocytes [89]. These revealed mechanosensitive genes in the MAPK pathway, alternatively spliced exons and mechanotransduction pathways involving different amino acid–base RNA binding proteins in myoblasts [88] and over 100 differentially regulated proteins in astrocytes after the application of mechanical strain [89].

Further, the studies described so far have mainly been 2D monolayers and 3D hydrogel encapsulated cells submerged in medium established in the Flexcell [31]. This makes it difficult to establish air liquid interface models on the Bioflex and tissue train membranes to investigate the effects of strain on physiologically relevant surfactant and/or mucus producing differentiated pulmonary epithelium. The use of specialized plates that allow for the co-culture of epithelial cells grown on transwell inserts (which can be used for ALIs) with other cell types cultured and stretched on the flexible membrane is a potential option to address this issue. Cells cultured on the flexible membrane experience direct effects of strain and interact with the epithelium which enable strain to indirectly regulate its phenotype through mechanotransduction pathways. This opens the potential to establish more multicellular systems as demonstrated by Sundarakrishnan and colleagues [77] including immune-fibroblast co- and tri- cultures. In line with this, multicellular models such as lung organoids which are self- assembling structures that form from lung epithelial progenitor cells with or without mesenchymal cell support, can be established on basement membrane proteins, harvested and incorporated in the Flexcell. As we previously alluded to in the section on the “Methods of applying strain on pulmonary cells” PCLS can also be strained through the application of pressure as is done with the Flexcell. These multicellular systems can then be combined with unbiased global transcriptomic, metabolomic and proteomic approaches such as single cell RNAseq [88], mass spectrometry [89] etc., to analyze the effect of the mechanodynamic environment on the representative models. Further to this, systems such as the MALI bioreactor where epithelial ALI’s directly experience strain, offer good alternatives in lung research when multicellular cultures are needed [31, 40]. These unique models can then enable the assessment of the lung mechanical environment’s effect on (aberrant) multicellular crosstalk in health and disease. We have demonstrated with SHG-NLOM that the structural organization of fibrillar collagen is disrupted in emphysematous chronic obstructive pulmonary disease (COPD) lungs [90]. In line with studies that have assessed fibroblast-ECM interactions in the lungs using the tissue train, it will be important for future work to mimic and assess disease pathobiology involving the alveolar tethering and lung subepithelial space using the tissue train linear and circular plates in combination with multicellular 3D hydrogel models and advanced imaging (e.g., SHG-NLOM & two-photon excitation fluorescence) to understand complex tissue remodeling mechanisms in conditions such as asthma, chronic cough and COPD. Added to this, there is also the opportunity to assess how exposure to noxious particles such as cigarette smoke, particulate matter from wildfire and pollution that are risk factors to lung diseases including asthma, COPD and IPF, affect cell–cell-ECM interaction in strain environments. Furthermore, as previously described in the section on different bioreactors, it is possible to interphase the Flexcell system with organs-on-chip, which enables the introduction of mechanical strain into biomimetic models that utilises microfluidic principles to incorporate fluid and airflow to mimic the lung (and gut) mucosa [41, 42]. It is therefore envisaged that the use of primary human cells, the introduction of multicellular models such as transwell co-cultures, interfacing the Flexcell with complex biomimetic organ-on-chip models and the use of MALI bioreactors will enable the discovery of mechanotransduction pathways hither-to under-studied or unknown. This will aid in further elucidating basic mechanisms of the mechanical environment in the healthy lungs and enable the discovery of new therapeutic targets for lung diseases.

## Conclusions

In this review, we presented an overview of studies that have utilized the Flexcell system to investigate the mechanodynamic environment of the breathing lung, with particular emphasis on fibroblasts in the healthy lung and different pathologies including ARDS, mechanical ventilation induced injury, BPD, pulmonary fibrosis, IPF, and asthma (Table 2). We found that a greater proportion of the mechanical models established in lung fibroblast Flexcell studies were 2D monolayer cultures set up in Bioflex plates with continuous human lung fibroblast cell-lines, in addition to primary animals and human lung fibroblasts. A few of the studies utilised lung-fibroblast-embedded collagen-I hydrogels in tissue train plates. The use of the Flexcell to apply mechanical strain of varying amplitudes as low as 1% to as high as 30% with frequencies from 0.1 – 2 Hz led to increased and decreased cell proliferation, ECM remodeling (resulting from aberrant ECM protein expression) as well as the regulation of inflammatory mediator release in lung fibroblasts. Hence these studies point to an important role of the mechanodynamic lung environment in regulating fibroblast phenotype and function with implications for the maintenance of the homeostatic lung environment and aberrant mechanisms in different lung diseases. These studies therefore point to an important role of the mechanodynamic lung environment in regulating fibroblast phenotype and function with implications for the maintenance of the homeostatic lung environment and aberrant mechanisms in different lung diseases. Further developments and research using more complex multicellular systems and global profiling techniques involving transcriptomics, metabolomics and proteomics may reveal hither-to understudied pathways and provide (new) therapeutic targets for (chronic) lung diseases.

## Data Availability

Not Applicable.

## Abbreviations

ARDS: Acute respiratory distress syndrome
BPD: Bronchopulmonary dysplasia
COPD: Chronic Obstructive Pulmonary Disease
ECM: Extracellular matrix
VILI: Ventilator-induced lung injury
ATII: Alveolar type II
PDMS: Poly(dimethylsiloxane)
FX: Flexcell
MALI: Moving air–liquid interface
IPF: Idiopathic Pulmonary Fibrosis
ALI: Air–liquid interface
2D: 2-Dimensional
3D: 3-Dimensional
LPS: Lipopolysaccharide
cpm: Cycles/minute
EGFR knockout: EGFR gene knocked out
PCNA: Proliferating cell nuclear antigen
TGF: Transforming growth factor
PHLFs: Primary human lung fibroblasts
MAPK: Mitogen-activated protein kinase
ERK: Extracellular signal-regulated kinase
JNK: C-Jun NH2-terminal kinase
MCP: Monocyte chemoattractant protein
RANTES: Regulated on activation normal T cell expressed and secreted
KC: Keratinocyte chemoattractant
TNF: Tumour necrosis factor
MIP: Macrophage inflammatory protein
HFL: Human fetal lung
MMP: Matrix metalloproteinase
Hz: Hertz
SHG-NLOM: Second Harmonic Generation Non-Linear Optical Microscopy
TPEF-microscopy: Two-Photon Excitation Fluorescence Microscopy
